# Exposure to Ideas, Evaluation Apprehension, and Incubation Intervals in Collaborative Idea Generation

**DOI:** 10.3389/fpsyg.2019.01459

**Published:** 2019-07-04

**Authors:** Xiang Zhou, Hong-Kun Zhai, Bibi Delidabieke, Hui Zeng, Yu-Xin Cui, Xue Cao

**Affiliations:** ^1^Department of Social Psychology, Nankai University, Tianjin, China; ^2^Department of International Business, School of Economics, Nankai University, Tianjin, China; ^3^Collaborative Innovation Center for China Economy, Tianjin, China

**Keywords:** exposure to ideas, evaluation apprehension, incubation intervals, idea generation, productivity deficits, group creativity

## Abstract

This study focused on the social factors and cognitive processes that influence collaborative idea generation, using the research paradigm of group idea generation, evaluation apprehension, and incubation. Specifically, it aimed to explore the impact of exposure to others’ ideas, evaluation apprehension, and incubation intervals on collaborative idea generation through three experiments. The results showed that in the process of generating ideas in a group, exposure to others’ ideas and evaluation apprehension can lead to productivity deficits in the number and categories of ideas, without affecting the novelty of ideas. Further, exposure to others’ ideas and evaluation apprehension had an interaction effect on the number of ideas. As compared with the situation without exposure to others’ idea, in that with exposure to others’ idea, evaluation apprehension had a weaker impact on the productivity of the number of ideas. Furthermore, incubation intervals were beneficial in reducing the negative effect of exposure to others’ ideas and in improving collaborative idea generation productivity.

## Introduction

Collaborative idea generation is a common phenomenon in organizations (Markman, 2016). According to this concept, when the group is regarded as a unit, members of the group will effectively gather the innovative resources and information they have mastered, and achieve deep collaboration and innovation within and between groups, through extensive interaction between members and resources (Sawyer and Dezutter, 2009; Hennessey and Amabile, 2010; Chen and Yang, 2012). The contemporary group creativity theories focus on the cognitive, social, and motivational factors that influence group performance on creativity. High-level group creativity requires an effective interactive process (Alper et al., 2007), optimal group composition (Zhang et al., 2016), positive group experience, and convenient backgrounds that support innovation and psychological security.

As the initial phase in group creativity, the quality and quantity of idea generation have a significant impact on later phases such as the selection and execution phases. Several studies have focused on the cognitive and social factors influencing performance on collaborative idea generation. In addition, researchers have also focused on developing strategies to promote productivity in idea generation. For instance, researchers have found that computer-supported electronic brainstorming and brain-writing can eliminate the negative effects of social inhibition on collaborative idea generation performance, alternating between face-to-face and electronic conference is an ideal choice for promoting collaborative idea generation (Paulus and Kenworthy, 2017). Korde and Paulus (2017) proposed that the hybrid brainstorming of individual and group innovation is an effective model for facilitating collaborative idea generation. After comparing the performance of interactive and nominal teams in collaborative learning groups using a task involving the generation of innovative ideas, Zhou et al. (2018) recommended promoting collective intelligence through strategic choices and rule setting in group activities, such as in collaborative learning.

The present study focused on the social factors and cognitive processes that influence collaborative idea generation, using the research paradigm of group idea generation, inducing evaluation apprehension and delayed incubation. It aimed to explore the impact of exposure to ideas, evaluation apprehension, and incubation intervals on group creativity through a series of experiments.

### Productivity Deficits in a Collaborative Idea Generation Task

Reporting that, when groups work together on idea generation tasks, they are less effective at creating ideas as compared to individuals, McGrath (1984) stated that “Individuals who work separately generate more creative and more ideas than working in groups, even after deleting the redundant part of their ideas.” Paulus et al. (1995) discovered that there is a “productivity deficit” in collaborative idea generation, and follow-up studies further explored the causes of such deficits.

The causes for productivity deficits may be rooted in the cognitive mechanism involved, including production blocking (Diehl and Stroebe, 1987), excessive demand for cognitive resources and working memory (Nijstad et al., 2003), fixation (Smith and Blankenship, 1989, 1991), and effects of part-list cueing inhibition and output interference (Rundus, 1973; Raaijmakers and Shiffrin, 1981). Diehl and Stroebe (1987, 1991) found evidence of production blocking in brainstorming. The main cause of production blocking is that multiple group members could not speak at the same time. Specifically, in their experiment, the “non-blocking” group allowed its members to speak at any time, and they reported that the nominal and “non-blocking” group generated more ideas than did the exposed group, and there was no difference between the nominal and “non-blocking” groups. Paulus and Yang (2000) found that the use of improved brainstorming, which allows group members to process fewer ideas each time, can increase productivity. These findings suggest that cognitive overload or long delays between idea generation and convergence can reduce productivity when a group is in the process of generating ideas (Nagasundaram and Dennis, 1993; Hinsz et al., 1997; Nijstad et al., 2003).

Evaluation of ideas and concerns about social activities may also lead to a decline in group performance. The social comparison process may lead individuals in the group to develop low-performance standards, leading to poor performance (Paulus et al., 2002). Further, the social comparison would cause social loafing. Since the responsibility in the process of completing group tasks is decentralized, if a group member feels that his/her contribution is not noticeable and that other group members are performing their duties, this member may be prone to slackening, and thus, he/she would make fewer contributions. Besides, in the process of collaborative problem-solving or collaborative ideation, individuals often face evaluation apprehension (Diehl and Stroebe, 1987) and exposure to other group members’ ideas (Kohn and Smith, 2010), which may also affect the quality of ideation.

### Evaluation Apprehension and Exposure to Ideas

Evaluation apprehension occurs when “the fear of negative evaluations from other group members or external members prevents participants who are working in groups from presenting their more original ideas” (Diehl and Stroebe, 1987). That is, because people may worry about negative evaluations from group members, they will generate fewer ideas when working together. Evaluation apprehension has negative effects on group idea generation. Collaros and Anderson (1969) set up three experimental conditions. In the high-evaluation apprehension group, four group members were informed separately that the other three members were experts; in the low-evaluation apprehension group, four group members were informed separately that one member of their group was an expert; and the members in the control group did not receive any information about their group members. The results revealed that the control group members produced the largest number of ideas, and the group which was informed that all other group members were experts produced the fewest. A post-experimental survey showed that the more a person thinks that he/she is surrounded by experts, the more threats he/she perceives, which in turn prevents him/her from providing more ideas. Camacho and Paulus (1995) examined the presence of individual differences in evaluation apprehension. Using the Interactive Anxiety Scale, they divided participants into low and high anxiety groups. The group type (real and nominal groups) interacted with the degree of interaction anxiety (low and high). Additionally, they found that, in real groups, low interaction anxiety level significantly increased the number of ideas generated. In addition, high-anxiety participants expressed hesitation in expressing their own ideas during the idea generation task. Thus, it was concluded that evaluation apprehension can lead to group productivity deficits and that it is better to choose a group of members with low interaction anxiety to avoid decrease in productivity.

Being exposed to other group members’ ideas also influences the collaborative idea generation. However, researchers hold different opinions about whether it has negative or positive effects. Fink et al. (2012) investigated the effects of exposure to others’ ideas on the originality of generated ideas using functional magnetic resonance imaging (fMRI). Their results suggested that being exposed to common or moderately creative ideas was effective in improving creativity. In contrast, Smith and Linsey (2011) purported that seeing or hearing other members’ ideas in a group would prevent individuals from contributing their own ideas. Though being exposed to others’ ideas would inspire individuals, there is also a disadvantage. Specifically, after hearing other group members’ ideas, individuals may tend to focus on or limit their ideas to similar categories. Consistent with Smith’s opinions, the present study assumed that being exposed to others’ ideas may reduce collaborative idea generation productivity.

Though many pieces of research have examined the impact of evaluation apprehension and exposure to ideas on collaborative creativity, fewer studies have focused on the interaction between these variables. The theory of social impact (Latané, 1981) states that the strength of some social powers, such as evaluation, is negatively relevant to the number of individuals addressed as the target of the social force (Henchy and Glass, 1969). Therefore, we could suppose that, as compared with participants who cannot see other group members’ ideas, those who can see others’ ideas will be clearly aware of the existence of others, leading to a reduction in the level of evaluation apprehension. McGrath (2015) found that compared with individuals, pairwise group structuring significantly reduced evaluation apprehension within idea generation groups. Given the above evidences, exposure to others’ ideas could reduce the negative impact of evaluation apprehension on group idea generation.

### Reduce Productivity Deficits: Incubation Effects

Incubation effects is an important branch of creativity research. The discussion on incubation effects stems from an interesting phenomenon. When facing an extremely difficult problem that we cannot solve immediately, we tend to put it on the shelf for a moment. Amazingly, we can solve the problem when we return to it after a break. The concept of incubation emerged in the 1920s, as a method or procedure of leaving the problem aside to solve it.

One of the most frequently and widely used paradigms in incubation studies is the delayed incubation paradigm (Gilhooly et al., 2012). In this paradigm, the experimental group is asked to perform “the aiming task (Stage 1)—incubation phase (interference task or relaxation)—the aiming task (Stage 2),” while the control group solves the problem in a single continuous phase. Paulus et al. (2006) set three experimental conditions to examine the effects of rest on individual brain-writing. A group of participants engaged in a 15-min idea generation task, a 6-min relaxation, and a 15-min idea generation task. Another group of participants engaged in a 10-min idea generation task, a 3-min relaxation, a 10-min idea generation task, a 3-min relaxation, and a 10-min idea generation task. Participants in the no-rest condition engaged in a 36-min idea generation task. On comparing group performances in the last 10 min in three conditions, researchers found that participants in the rest conditions produced more ideas than did those in the no-rest condition. However, there was no difference between the two experimental groups with rest intervals. In addition, Lu et al. (2017) found that in creativity tasks that require divergent or convergent thinking, constant interference task could increase creativity by reducing cognitive fixation. Though adopting the same paradigm, previous studies focused on various aspects of the incubation period, such as the target problems, the types of the interpolated tasks and the span of the incubation period (Sio and Ormerod, 2009).

Smith and Blankenship (1991) proposed the selective forgetting hypothesis to account for incubation effects, suggesting that an incubation period provides time for forgetting the thoughts that are not helpful or even detrimental to the problem-solving, and in turn, problem solvers will be less sensitive to these irrelevant concepts. Thus, they can have a fresh perspective toward the current problem and generate new solutions on it (Segal, 2004). As a result, if an individual is stuck on a fixed path while solving a specific problem and cannot generate more ideas, incubation intervals may help improve the situation, reduce productivity deficits, and facilitate problem-solving (Penney et al., 2011).

This study intended to investigate the independent and interaction impacts of evaluation apprehension and exposure to ideas on collaborative idea generation. Evaluation apprehension and exposure to other people’s ideas are both important factors that affect creativity. Both stem from the other people’s perceptions and are related to the dual processing mechanism of emotion and cognition. Subsequently, we attempted to identify out whether incubation intervals can increase productivity in collaborative idea generation, and if so, how different incubation intervals affect collaborative idea generation. In addition, dyads are sufficiently small groups to provide cognitive stimulation (such as exposure to others’ ideas) while not raising the fear of evaluation from group members to impairing level (Brown et al., 1998; Nijstad and Stroebe, 2006). Therefore, all the groups in this study were in the form of dyads in order to exclude the irrelevant variables.

## Experiment 1

Experiment 1 explored the impact of evaluation apprehension and exposure to others’ perspectives on creative idea output in groups. We aimed to examine the main effect of evaluation apprehension and exposure to others’ ideas, and accordingly proposed the following hypotheses:

Hypothesis 1: Compared with the groups in the exposed condition, those in the non-exposed condition would perform better on a collaborative idea generation task.

Hypothesis 2: Compared to the condition with evaluation apprehension, groups would perform better on a collaborative idea generation task in the condition without evaluation apprehension.

In addition, we purported Hypothesis 3 based on the social impact theory mentioned above.

Hypothesis 3: There is an interactive effect between evaluation apprehension and exposure to group members’ ideas on group idea generation task performance, and this exposure will weaken the negative impact of evaluation apprehensions.

### Method

#### Participants

We recruited participants on the communication platform of universities, 178 college students were recruited for this experiment. A total of 89 men and 89 women are recruited and formed 89 groups, each group had one man and one woman. Participants’ age ranged from 19 to 23 years. All the groups were divided into one of four experimental conditions randomly. Excluding participants whose answer was blank or did not fit the subject, valid data were collected from 80 dyads. None of the participants had participated in any similar experiment in the past, and they were paid after the present experiment.

#### Design and Procedure

The experiment employed a 2 (exposure to ideas: exposed vs. non-exposed) × 2 (evaluation apprehension: present vs. absent) design. Participants were randomly assigned to one of the following four experimental conditions: exposed-evaluation apprehension-present group, exposed-evaluation apprehension-absent group, non-exposed-evaluation apprehension-present group, non-exposed-evaluation apprehension-absent group.

In the exposed group, participant dyads were instructed to complete an idea generation task by submitting ideas together to experimenters *via* WeChat. Participants within dyads could see group member’s ideas but they were not allowed to communicate with each other. In the non-exposed group, each participant completed the idea generation task by submitting ideas individually to experimenters *via* WeChat. They could not see others’ opinions or communicate with them. Before the experiment, the exposed and non-exposed groups were informed that they would generate ideas with another person in a dyad.

In the evaluation apprehension condition, participants were informed that other participants would evaluate their ideas at the end of the experiment. In the evaluation apprehension absent condition, they were informed that the quality of their ideas would not be judged.

Before the commencement of the experiment, participants received explanations to introduce the experiment and the task (a topic of idea generation) using the following text: “Please list methods that can improve the university you study in.” In the next 20 min, participants sent their ideas to the experimenter *via* WeChat. They did not receive communication or feedback from the experimenter during the task. In the exposed group, participants could see the ideas provided by their group members.

#### Task and Measure

The topic of idea generation was “Please list methods that can improve the university you study in.” Similar topics have been used in other studies on creativity (Marsh et al., 1997; Paulus et al., 2006; Putman and Paulus, 2009; Baruah and Paulus, 2011). WeChat was used as the tool for experiment operation and idea submission.

As for measures, based on the encoding indexes proposed by Kohn and Smith (2010), experimenters drew a revised set of encoding indexes that included 30 categories. For instance, “course” may include “more diverse curriculum settings”; “diet” may include “regular check-ups in the school cafeteria.” Two scorers were invited to divide the 2,159 items generated by 160 participants into 30 categories (see Appendix). Scorers were trained before the rating phase to ensure that they understood the meaning of and criteria for each category adequately. In the rare condition that participants reported more than two ideas for one item, experimenters would divide and classify this item appropriately (e.g., “train teachers” and “install an air conditioner in the dormitory” would be respectively be encoded to “teacher” and “dormitory” categories). Because the coding results are nominal variables, we introduced category agreement (CA) and intercoder reliability coefficient to calculate the inter-rater reliability. CA refers to the proportion of the consistent ideas. And the intercoder reliability coefficient (*R*) = (n − 1) × CA/(1 + n × CA). In experiment 1, CA = 0.68, *R* = 0.81. When the two coders were divided on the classification, they were asked to discuss the issue until reaching an agreement. Repeated or unserious ideas were excluded from the data analysis.

When creating the non-exposed group, experimenters randomly assigned data from two participants in the nominal group to one set and arranged these ideas chronologically. The novelty level of ideas was determined using the following formula: novelty score of Category *X* = (total number of ideas/number of ideas falling into Category *X*)/(total number of ideas/100) (Kohn and Smith, 2010). As a result, the fewer the number of ideas in a category, the higher was its novelty score.

#### Results and Discussion

We conducted a two-way analysis of variance (ANOVA) on the number, category, and novelty of ideas generated by participants from different evaluation apprehension and exposed conditions.

**Figure 1:**
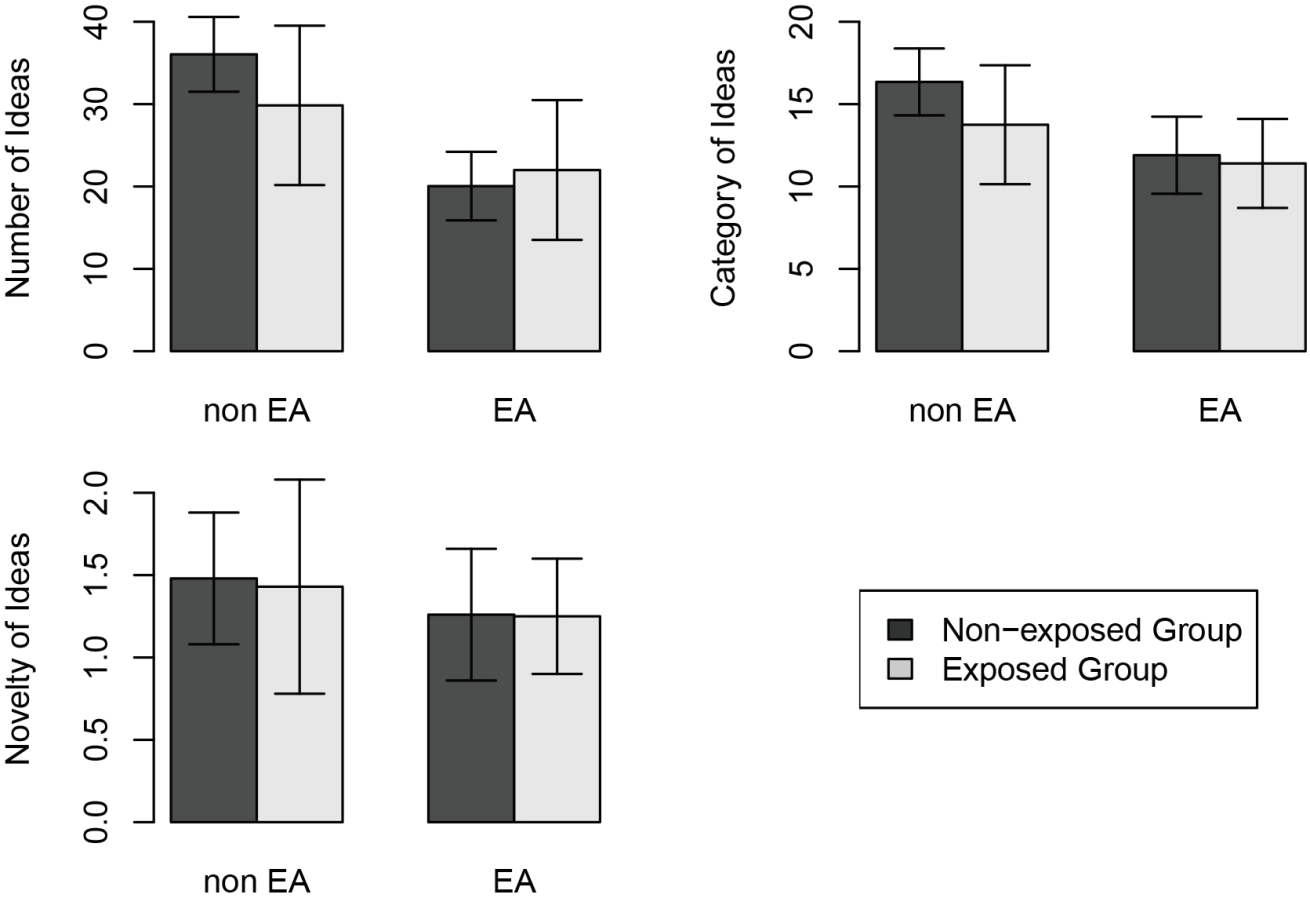
Group idea generation under four conditions (the error bars refer to standard error).

When it came to the number of ideas, there was a main effect of evaluation apprehension [*F*(1, 76) = 55.876, *p* < 0.001, *η*^2^ = 0.424, 1 − *β* = 0.98], with the evaluation apprehension-absent group (*M* = 32.95, SD = 8.17) producing more ideas than evaluation apprehension-present group (*M* = 21.03, SD = 6.76). The main effect of idea exposure was not significant [*F*(1, 76) = 1.774, *p* > 0.1]. There was a significant interaction between evaluation apprehension and idea exposure [*F*(1, 76) = 6.525, *p* = 0.013, *η*^2^ = 0.079, 1 − *β* = 0.47]. The simple effect analysis revealed that non-exposed groups (*M* = 36.05, SD = 4.54) generated more ideas than exposed groups (*M* = 29.85, SD = 9.68) under the evaluation apprehension-absent condition. But under the evaluation apprehension-present condition, the number of ideas generated by non-exposed groups (*M* = 20.05, SD = 4.16) and exposed groups (*M* = 22.00, SD = 8.49) were almost the same (see Figure 1).

Regarding idea categories, the main effect of evaluation apprehension was significant [*F*(1, 76) = 30.886, *p* < 0.001, *η*^2^ = 0.289, 1 − *β* = 0.94], with the evaluation apprehension-absent group (*M* = 15.05, SD = 3.20) generating more categories of ideas than evaluation apprehension-present group (*M* = 11.65, SD = 2.53). The main effect of exposure to other’s ideas on categories was also significant [*F*(1, 76) = 6.419, *p* = 0.013, *η*^2^ = 0.078, 1 − *β* = 0.47], with the non-exposed group (*M* = 14.13, SD = 3.12) generating more categories of ideas than exposed group (*M* = 12.58, SD = 3.37). Whereas, the interaction between evaluation apprehension and exposure to ideas was not significant [*F*(1, 76) = 2.946, *p* > 0.05].

As for the novelty of ideas, both the main effects of evaluation apprehension [*F*(1, 76) = 3.566, *p* > 0.05] and exposure to ideas [*F*(1, 76) = 0.091, *p* > 0.1] and their interaction effect [*F*(1, 76) = 0.035, *p* > 0.1] were not significant.

As shown above, exposure to others’ ideas significantly weakened the idea category diversity, which partly supported Hypothesis 1. Possibly, participants were influenced by the ideas expressed by other group members, which may have led to the occurrence of fixation. Specifically, participants may have paid too much attention to others’ ideas, which would have in turn limited their views to a small number of categories, leading to the deficit in their productivity in terms of categories of ideas generated during collaborative idea generation.

It was also observed that participants under evaluation apprehension condition generated fewer ideas and explored fewer categories as compared to participants under evaluation apprehension absent condition, suggesting that participants’ expression of ideas was hindered by the presence of evaluation apprehension. In the evaluation apprehension condition, participants tended to adopt a more conservative strategy for idea generation. That is, they may have avoided broadening the categories of ideas or putting forward more original ideas to avoid receiving a negative evaluation. Hypothesis 2 was supported.

The results also revealed that there was a significant interaction between evaluation apprehension and idea exposure on the number of ideas, which partly supports Hypothesis 3. Specifically, in the absence of evaluation apprehension, the exposed group performed more poorly as compared to the non-exposed group in terms of the number of ideas. However, in the presence of evaluation apprehension, the difference between the exposed group and the non-exposed group was not significant anymore. It suggested that the idea generation task performance of exposed and non-exposed groups both decreased under apprehension condition, but the degree of decline in the non-exposed group’s performance was higher than in the exposed group. This result indicates that, as compared with the non-exposed condition, when the exposure of ideas was present, evaluation apprehension had a less negative influence on productivity in terms of the quantity of collaborative idea generation. This finding supported Hypothesis 3.

## Experiment 2

Based on Experiment 1, Experiment 2 explored methods to eliminate the negative effect of exposure to others’ ideas on idea generation. As shown in previous research, in creative problem-solving and memory retrieval, incubation intervals could be used to relieve fixation (Browne and Cruse, 1988; Smith and Blankenship, 1989). Relaxation or interference tasks (Lu et al., 2017) during the process of idea generation could promote individual or group behavior in an idea generation meeting. Therefore, Experiment 2 tried to provide two experimental settings (relaxation and interference task) to explore whether incubation can reduce the negative impact of exposure to others’ ideas, thus improving collaborative creativity.

### Method

#### Participants

A total of 98 school students were recruited from different universities across the country. None of the participants had participated in Experiment 1 or any similar experiment before. Participants were paid after the experiment. They were divided into 49 dyads, each dyad containing one male and one female. Because one dyad did not understand the rules clearly, data collected from the remaining 48 dyads were used for the analysis.

#### Design and Procedure

Participants were randomly divided into the following three groups: exposed-immediate group, exposed-relaxation group, and exposed-task group. Each group had 16 dyads. The procedure was similar to that of Experiment 1. Participants in the above three exposed groups were asked to complete the idea generation task by submitting ideas together to experimenters *via* WeChat. They could see group members’ ideas, but they could not communicate with each other. In the exposed-immediate group, participants were required to conduct the idea generation task for 20 continuous minutes without a break. In the exposed-relaxation group, participants were required to stop the idea generation task after 10 min and to relax and not conduct any other activities. In the exposed group, participants were given a break to complete other cognitive tasks after a 10-min idea generation task. After 5 min, participants were required to continue conducting the idea generation task, and they could not submit any ideas that had been generated before. After 10 min, the second idea generation task ended.

#### Task and Measure

Like in Experiment 1, the topic of idea generation was “List ways to improve your present university.” WeChat was used as a tool for experiment operation and idea submission. Experimenters collected ideas from participants in the first and last 10 min of the experiment and classified them into 30 categories using the method employed in Experiment 1 (CA = 0.65, *R* = 0.79). Finally, they calculated the number of categories and originality of ideas generated by each dyad.

The interference tasks used in this experiment were 10 graphics and logical reasoning questions extracted from the Civil Servant Test Bank (assessment method of the civil servant in China). In order to avoid the extra negative emotion that might come with interference tasks, the questions would not be scored, and the correct answer would be provided after each question was completed. The interference tasks were just used to let the subject break from the original task temporarily.

#### Results and Discussion

In order to test the differences of idea generation performance under different conditions, three ANOVAs were respectively conducted in the first 10 min, the last 10 min and the total 20 min of the process. Since the present study was conducted to test three independent hypotheses on the same data set, the negative interval in this study was set as *α*/3 *via* Bonferroni correction.

An ANOVA on the number, category, and novelty of ideas generated by participants in different groups showed that, in the first 10 min and the total 20 min, there were no significant differences in the number, category, and novelty of ideas generated by the dyads.

In contrast, in the last 10 min, the number of ideas generated by the dyads in the three conditions were significantly different, [*F*(2, 45) = 5.243, *p* < 0.05/3, *η*^2^ = 0.189, 1 − *β* = 0.807], and the difference among three conditions on the categories of ideas was marginally significant, [*F*(2, 45) = 4.350, *p* < 0.1/3, *η*^2^ = 0.162, 1 − *β* = 0.725]. However, the difference in the novelty of ideas generated by participants among the different groups was not significant [*F*(2, 45) = 1.693, *p* > 0.1]. Furthermore, *post hoc* multiple comparisons showed that the dyads in the two kinds of incubation conditions generated significantly more ideas and their ideas had more categories than did those in the immediate condition (see Table 1). On applying the Bonferroni correction, the cutoff for the significance level was 0.017, and the difference in the quantity of ideas (mean difference (MD) = 4.81, *p* = 0.003) and categories (MD = 2.75, *p* = 0.009) between the exposed-task and exposed-immediate groups was still significant, while that in the difference in the number of ideas (MD = 3.50, *p* = 0.028) and categories (MD = 2.31, *p* = 0.026) between the exposed-relaxation and exposed-immediate groups was not significant anymore.

**Table 1:** *Post hoc* multiple comparisons of ideas generated in last 10 min (Experiment 2).

Time	*I*	*J*	MD	*p*
Number	Immediate	Relax	−3.50	0.028
		Task	−4.81	0.003
	Relax	Task	−1.31	0.398
Category	Immediate	Relax	−2.31	0.026
		Task	−2.75	0.009
	Relax	Task	−0.44	0.664

The results indicated that both kinds of incubation interventions reduced the negative effects of exposure to others’ ideas on the group’s creative productivity. Additionally, it was suggested that relaxing and interference task during the incubation interval could promote idea generation by having participants temporarily take their attention away from the present task, thus reducing their cognitive fixation.

## Experiment 3

Experiment 1 and 2 did not control the contents and number of examples that participants could see. Therefore, the member in exposed groups could see the other group member’s opinions. This kind of experimental setting possesses certain ecological validity. However, because the process of idea generation was not controlled by the experimenter, it was necessary to conduct a more rigorous experiment to verify the results of Experiment 1 and 2, including the negative effects of the process of generating innovative ideas, and the effect of incubation on these negative effects. Therefore, Experiment 3 controlled the examples of the ideas presented to participants, to further verify whether the two incubation methods (relaxation and interference task) could weaken the negative effects of exposure to other people’s opinions during collaborative idea generation.

### Method

#### Participants

A total of 111 school students were recruited from different universities across the country. There were 34 men and 77 women, with age ranging from 18 to 26 years. None of the participants had participated in Experiment 1 or 2, or any similar experiment before. Participants were paid after the experiment. After excluding invalid data, the final effective sample size was 104.

#### Design and Procedure

Participants were randomly divided into the following four groups: non-exposed group (control group), exposed-immediate group, exposed-relaxation group, exposed-task group. Each group comprised of 26 participants. Participants were asked to complete idea generation tasks individually *via* WeChat with the experimenter. In three exposed groups, participants received ideas from the experimenters in the first, third, fifth, and seventh minute of the process of idea generation. Additionally, they were informed that the examples were from their group member. In fact, the examples came from the four most frequently appearing categories in Experiment 1. Each example presented one category, along with three other alternatives. If the idea had been put forward by the participant him/herself, alternative ideas were used. Participants in the non-exposed (control) group did not receive any ideas from the experimenter.

The manipulation of incubation was similar to that used in Experiment 2. In the control and exposed-immediate groups, participants performed the idea generation task for 20 continuous minutes, without breaks. In the exposed-relaxation group, participants were required to stop the idea generation task after 10 min, and to relax and not conduct any other activities. In the exposed-task group, after a 10-min idea generation task, participants were given a break to complete other cognitive tasks. After 5 min, participants in the two incubation conditions were required to continue conducting the original idea generation task, and they could not submit any ideas that had been generated before. After 10 min, the second idea generation task ended.

#### Task and Measure

As in Experiment 1 and 2, the topic of idea generation was “List ways to improve your present university.” WeChat was used as a tool for experiment operation and idea submission. Experimenters collected ideas from participants in the first and last 10 min of the experiment and classified them into 30 categories using the method employed in Experiment 1 and 2 (CA = 0.72, *R* = 0.83). Finally, they calculated the number of categories and originality of ideas generated by each participant.

#### Results and Discussion

In order to test the differences of idea generation performance under different conditions, three ANOVAs were respectively conducted in the first 10 min, the last 10 min, and the total 20 min of the process. Since the present study was conducted to test three independent hypotheses on the same data set, the negative interval in this study was set as *α*/3 *via* Bonferroni correction.

An ANOVA on the number, category, and novelty of ideas generated by the participants in the different groups showed that, in the first 10 min, there were no significant differences in the number [*F*(3,100) = 0.597, *p* > 0.1], category [*F*(3, 100) = 0.859, *p* > 0.1] and novelty [*F*(3, 100) = 1.326, *p* > 0.1] of ideas generated by participants.

In the last 10 min (see Table 2), the number [*F*(3, 100) = 5.06, *p* < 0.01/3, *η*^2^ = 0.132, 1 − *β* = 0.909] of ideas generated by participants in the last 10 min differed significantly across the four conditions. Furthermore, *post hoc* multiple comparisons (with Bonferroni correction, the cutoff for the significance level was 0.008) showed that participants in the exposed-immediate group generated fewer ideas as compared to the number of ideas generated by participants in the exposed-relaxation group (MD = −2.483, *p* < 0.001), exposed-task group (MD = −1.751, *p* = 0.010, marginal significant) and non-exposed group (MD = −1.789, *p* = 0.009, marginal significant), while the difference in the number of ideas among exposed-relaxation group, exposed-task group, and non-exposed group was not significant.

**Table 2:** *Post hoc* multiple comparisons of ideas generated in last 10 min (Experiment 3).

	*I*	*J*	MD	*p*
Number	Non-exposed	Exposed-immediate	1.79	0.009
		Exposed-relax	−0.69	0.292
		Exposed-task	0.04	0.954
	Exposed-immediate	Exposed-relax	−2.48	0.000
		Exposed-task	−1.75	0.010
	Exposed-relax	Exposed-task	0.73	0.266
Category	Non-exposed	Exposed-immediate	1.10	0.020
		Exposed-relax	−0.58	0.209
		Exposed-task	0.12	0.803
	Exposed-immediate	Exposed-relax	−1.68	0.000
		Exposed-task	−0.99	0.036
	Exposed-relax	Exposed-task	0.69	0.132

As for idea categories, the results of ANOVA revealed that it differed significantly across the three conditions [*F*(3, 100) = 4.55, *p* < 0.05/3, *η*^2^ = 0.120, 1 − *β* = 0.874]. *Post hoc* multiple comparisons showed that participants in the exposed-immediate group explored fewer idea categories as compared to the category of ideas generated by participants in the other conditions. After Bonferroni correction, only the difference between exposed-immediate group and exposed-relaxation group was still significant (MD = 1.680, *p* < 0.001).

Besides, the difference in the novelty of ideas generated by participants among the four groups was not significant [*F*(3, 100) = 2.43, *p* > 0.1].

An inter-group ANOVA on the number, category, and novelty of ideas in the total 20 min showed that there was a marginally significant difference in the number of ideas generated among four conditions [*F*(3, 100) = 3.283, *p* < 0.1/3, *η*^2^ = 0.090, 1 − *β* = 0.736]. Furthermore, *post hoc* multiple comparisons showed that, after Bonferroni correction, participants in the exposed-relaxation condition generated significantly more ideas than did those in the exposed-immediate group (MD = 3.210, *p* = 0.003). The results of ANOVA are listed in Table 3.

**Table 3:** *Post hoc* multiple comparisons of ideas generated in total 20 min (Experiment 3).

	*I*	*J*	MD	*p*
Number	Non-exposed	Exposed-immediate	1.61	0.136
		Exposed-relax	−1.60	0.131
		Exposed-task	−0.77	0.470
	Exposed-immediate	Exposed-relax	−3.21	0.003
		Exposed-task	−2.38	0.029
	Exposed-relax	Exposed-task	0.83	0.431

## Discussion

One of the goals of this paper is to further explore the comprehensive impact of opinion exposure and evaluation apprehension on the creation of collaborative ideas. The result of Experiment 1supported the main effect and the interaction effect of exposure to others’ ideas and evaluation apprehension in group idea generation. We also tried to explore whether two different incubation methods (relax/task conversion) had a significant effect on reducing the negative impact of exposure on group creativity and thus improving idea generation productivity.

### Impact of Evaluation Apprehension and Exposure to Others’ Ideas

Experiment 1 revealed that exposed groups generated fewer ideas than did non-exposed groups, and their ideas included fewer categories. This result may have appeared because, in the process of social interaction in the cooperative innovation group, if members saw the ideas of other members, they would focus too much attention on others’ ideas, thus limiting their scope of thinking and reducing the output of innovative ideas.

In this study, to elicit evaluation apprehension among participants, the experimenter’s instructions prompted participants to conduct external evaluations. The main effects of this manipulation were significant. Further, in the presence of evaluation apprehension, the number and categories of ideas generated by the non-exposed and exposed groups decreased. This finding is consistent with those of previous research, and it evidences the presence of the negative impact of evaluation apprehension on the group’s innovative idea generation. It is worth mentioning that the evaluation apprehension and exposure to others’ ideas had an interaction effect on the number of categories of ideas generated. This phenomenon can be explained using the social impact theory mentioned earlier in this manuscript. When the evaluation expectation is conducted in exposed groups, the presentation of other members’ answers clearly conveys their existence, thus virtually sharing the pressure of this evaluation. This, in turn, weakens the negative impact of the evaluation apprehension. Therefore, compared with non-exposed groups, the reduction in innovation productivity was less severe in the presence of evaluation apprehension in exposed groups.

### Role of Incubation Interval

Experiment 2 explored possible measures to reduce the negative effects of exposure and to improve groups’ creative output by introducing two different incubation methods. By controlling the exposure to ideas received by participants and simulating a collaborative situation in the creative generation task, Experiment 3 conducted a more rigorous experiment to verify the results of Experiment 1 and 2.

The results of Experiment 2 showed that both incubation methods increased the number and categories of ideas generated by dyads in the last 10 min, supporting the positive effect of incubation in collaborative idea generation. The difference between the two methods was not significant, which means that putting the problem aside and relaxing or performing other tasks both promoted the generation of innovative ideas. This result could be explained by selective forgetting hypothesis (Smith and Blankenship, 1991). Participants’ attention would be temporarily diverted when led to a break or interference task, contributing to forgetting the irrelevant or redundant ideas from the previous task. Thus, participants may generate ideas from different perspectives after the incubation period.

In Experiment 3, there was no significant difference in the number, category, and novelty of ideas generated by the exposure and control groups in the first 10 min among four conditions. This may indicate that participants’ ideas were not affected by exposure to others’ ideas immediately. Despite this, its role in the inhibition of innovative idea generation appeared as the experimental time increased, especially in the last 10 min. A comparison of the performance of the four groups of participants in the last 10 min revealed that the control, exposure-relaxation, and exposure-task groups produced more ideas than the exposure-immediate group, and their ideas included more categories, suggesting the number and types of ideas produced were affected negatively by exposure to others’ ideas, and this effect was more likely to manifest later in the task. On the other hand, the present study found that the incubation interval had a positive impact on the number and type of ideas generated by participants, which is consistent with the results of Experiment 2 and findings of previous studies (Penaloza and Calvillo, 2012).

The number and types of ideas generated by the two incubation groups were higher than those of the exposure-immediate group in the total 20 min, but not significantly. This may be due to the fact that the experiment was used for thinking and suggesting that the experiment was only 20 min long, and cannot reflect enough utility. Subsequent research can consider increasing the length of time to continue to explore the effects of incubation.

## Conclusion

In our daily life, collaborative idea generation is a widely used working technique, which is often applied to the conceptual design group (Sutton and Hargadon, 1996), the research team and various other forms of collaborative groups. However, the productivity loss caused by collaborative idea generation is also an ongoing concern of researchers. Based on the results of this study, we speculate that when individuals work in a group, they will: (1) pay too much attention to evaluation when surrounded by people, resulting in evaluation apprehension and (2) tend to conform to other group members, as well as fixate their ideas on the existing ideas of others. Both of the points above will limit individuals’ creativity and reduce the number and categories of ideas. This kind of influence will finally result in productivity deficits, which means that when people work together, they will generate fewer ideas than work alone. Furthermore, this study also tended to explore effective strategies to weaken the negative effects. According to the results of Experiment 2 and Experiment 3, we draw support from previous studies that relaxation and interference task in the incubation interval significantly diminish the negative effects of exposure to other people’s ideas.

In summary, this study focused on the social factors and cognitive processes that influence collaborative idea generation using the research paradigm of group creativity generation, evaluation of concern induction, and incubation paradigm. Additionally, this study explored the impact of perspective exposure, evaluation apprehensions, and incubation intervals on cooperative innovation productivity through three experiments. The results showed that the second half of the group’s idea generation with exposure and evaluation apprehension led to a reduction in the number and type of ideas generated by the groups. Further, these factors had an interaction effect on the number of creative ideas generated. Compared to that in participants with no exposure, in the case of exposure, the impact of evaluation apprehension on the quantitative productivity of cooperative innovation ideas was weak. The addition of incubation intervals helped to increase innovation productivity, suggesting that relax and task transitions are beneficial for reducing the negative impact of exposure on idea generation.

## Limitations And Prospects

Although this study explored the role of exposure to others’ opinions, evaluation apprehensions, and incubation intervals on the generation of group innovation ideas, it had some limitations. For instance, the example ideas presented to participants in Experiment 3 were the most typical types of ideas that emerged in the idea generation task in Experiment 1. Therefore, future researchers can further explore the impact of presentation of typical and atypical, or novel and non-innovative examples. Thus, the scalability of idea presentation can be strengthened further. In addition, novelty score was not significantly different on different levels of idea exposure nor evaluation apprehension. The probable explanation may be that the scoring rule was not fully appropriate. And in future studies, we could consider changing the scoring rules. Furthermore, this study used two-person groups. Future research can further explore incubation effects on groups of different sizes and explore the effects of length of incubation interval, length of thinking in the task, and arrangement of incubation intervals, to generate more creative strategies to improve subsequent productivity of collaborative idea generation groups. The impact of the arrangement of activities during the incubation period is also worth exploring. For example, the task form and complexity of the interpolation task are areas worth exploring.

## Ethics Statement

This study was carried out in accordance with the recommendations of the ethical review work guidelines of the Institutional Review Board of Psychology of the Nankai University with written informed consent from all subjects. All subjects gave written informed consent in accordance with the Declaration of Helsinki. The protocol was approved by the Institutional Review Board of Psychology of the Nankai University.

## Author Contributions

XZ, H-KZ, BD, Y-XC, and HZ developed the research idea together. Under the supervision of XZ, BD, H-KZ, and Y-XC collected and analyzed the data for Experiment 1, 2 and 3. XZ, BD, and XC drafted the manuscript. HZ, H-KZ, and Y-XC provided critical revisions.

### Conflict Of Interest Statement

The authors declare that the research was conducted in the absence of any commercial or financial relationships that could be construed as a potential conflict of interest.

## Appendix: The Category List of Idea Generation

**Table:**

Expansion	Practice	Classroom
Investment	Exchange	Library
Publicity	Environment	Traffic
Culture	Equipment	Food
Discipline construction	Dormitory	Service
Admission	Information	Energy
Course	Safety	Activity
Teacher	Cost	Health
Employment	Administration	Sports
Academic	Advice	Network
